# High-dose versus standard-dose intermittent meropenem in critically ill patients: An observational cohort study

**DOI:** 10.1016/j.aicoj.2025.100022

**Published:** 2026-01-16

**Authors:** Felix Bergmann, Marlene Prager, Lena Pracher, Katarina Kumpf, Markus Zeitlinger, Anselm Jorda

**Affiliations:** a Department of Clinical Pharmacology, Medical University of Vienna, Vienna, Austria; b Division of Infectious Diseases and Tropical Medicine, Department of Medicine I, Medical University of Vienna, Vienna, Austria; c IT Systems and Communications, Medical University of Vienna, Vienna, Austria

**Keywords:** Sepsis, Antimicrobial, Antibiotic, Pneumonia, Pharmacokinetics

## Abstract

**Background:**

The impact of high-dose versus standard-dose meropenem on outcomes in critically ill patients remains uncertain.

**Methods:**

We conducted an observational cohort study in Vienna, Austria, including critically ill patients treated with meropenem from March 2014 to March 2024. Eligible patients had an intensive care unit (ICU) stay of ≥3 days and received either high-dose (6 g/day) or standard-dose (3 g/day) meropenem via intermittent infusion (or equivalent doses in patients with impaired renal function). We applied 2:1 propensity score matching with covariate adjustment to adjust for confounding by indication. The primary outcome was 90-day all-cause mortality. Secondary outcomes included 30-day mortality, emergence of antimicrobial resistance, initiation of extracorporeal membrane oxygenation (ECMO), new onset of acute respiratory distress syndrome (ARDS) and length of ICU and hospital stay. Occurrence of acute kidney injury (AKI) was evaluated as a safety endpoint.

**Results:**

Of 4,210 critically ill patients who received meropenem, we matched 1144 treated with high-dose intermittent infusions with 572 patients who received standard-dose therapy. The 90-day all-cause mortality was significantly lower in the high-dose meropenem group (adjusted risk, 31.5%; 95% CI, 28.5–34.5) compared to the standard-dose group (adjusted risk, 40.9%; 95% CI, 36.7–45.1), with an adjusted risk difference of 9.4% (95% CI, 4.8–14.1; p < 0.001). Secondary outcomes did not differ between groups, with adjusted risks for 30-day mortality (high-dose vs. standard-dose: adjusted risks, 20.0% vs. 22.2%), resistance emergence (5.0% vs. 6.2%), ECMO initiation (6.1% vs. 7.3%), and new-onset ARDS (20.8% vs. 24.9%) showing no significant differences. Adjusted mean ICU and hospital length of stay were comparable (21.5 vs. 21.4 days and 44.3 vs. 41.7 days, respectively). High-dose therapy was associated with a lower adjusted risk of AKI (60.6% vs. 68.2%)

**Conclusions:**

High-dose intermittent meropenem was independently associated with lower 90-day all-cause mortality compared with standard-dose therapy in critically ill patients.

## Introduction

Severe infections remain among the leading causes of morbidity and mortality in intensive care unit (ICU) populations [1]. Early initiation of appropriate antimicrobial therapy is critical, as delays or inadequate coverage are strongly linked to increased mortality [2]. Meropenem is widely used as both empirical and targeted therapy for severe infections in critically ill patients. Its structural stability against many β-lactamases and favorable safety profile make it particularly valuable in critically ill populations, where multidrug-resistant (MDR) Gram-negative pathogens are increasingly prevalent [3]. However, critical illness is frequently accompanied by pathophysiological alterations in renal function, tissue distribution, and organ performance that may affect pharmacokinetics and thereby impair the efficacy of antimicrobials [4].

Previous studies have shown that conventional dosing regimens of 1 g every 8 h as intermittent infusions (3 g/day) may be insufficient to reach therapeutic pharmacokinetic/pharmacodynamic (PK/PD) targets in ICU patients [3,5]. PK simulations and clinical data suggest that higher doses of 2 g every 8 h, resulting in a total daily dose of 6 g, may increase target attainment and potentially improve outcomes [6,7].

Current Infectious Diseases Society of America (IDSA) and European Society of Clinical Microbiology and Infectious Diseases (ESCMID) guidelines support meropenem as first-line therapy for critically ill patients and advocate high-dose regimens (i.e., 2 g every 8 h) to improve outcomes [8,9]. In contrast, the Summary of Product Characteristics (SmPC) for meropenem generally recommends a total daily dose of 3 g (1 g q8h) for severe infections such as pneumonia, complicated urinary tract infections, and complicated skin and soft tissue infections [10]. Higher doses (up to 6 g/day) are reserved in regulatory labeling for bronchopulmonary infections in cystic fibrosis and for acute bacterial meningitis. This discrepancy between clinical practice guidelines and regulatory labeling underscores ongoing uncertainty regarding the clinical value and safety of high-dose regimens in broader ICU populations. This may be due to the fact that much of the available evidence supporting 6 g/day dosing derives from PK/PD modeling, Monte Carlo simulations, or small, infection-specific trials [[11], [12], [13], [14]].

At our institution, both high-dose (6 g per day) and standard-dose (3 g per day) meropenem treatment protocols are frequently used in critically ill patients with preserved renal function, regardless of the site or type of infection. This study therefore investigates whether such high-dose therapy provides clinical advantages over the conventional 3 g daily regimen in this population.

## Methods

### Study design and participants

This retrospective observational, propensity score matched, observational cohort study included adult patients (aged ≥18 years) receiving intensive care at the University Hospital of Vienna, associated with the Medical University of Vienna, Austria, from March 2014 to March 2024. We included patients with an ICU stay of at least three days which received either standard-dose or high-dose meropenem administered as intermittent infusions. Standard-dose treatment was defined as a total daily meropenem dose of 3 (±1) g in patients with normal renal function. High-dose treatment was defined in accordance with the SmPC, including 6 (±1) g/day in patients with normal renal function, 4 g/day in patients with an eGFR of 26–50 mL/min/1.73 m^2^, 2 g/day in patients with an eGFR of 10–25 mL/min/1.73 m^2^, and 1 g/day in patients with an eGFR <10 mL/min/1.73 m^2^. Daily doses were calculated by dividing the total administered amount by the number of treatment days. Patients who received both standard and high-dose meropenem were ultimately categorized based on their cumulative mean dose.

We defined the “index date” as the first initiation of meropenem therapy and each patient was included only once at their first treatment course with meropenem.

The study adhered to ethical standards according to the Declaration of Helsinki and received approval from the Ethics Committee of the Medical University of Vienna (reference number: EC 1348/2024). Patient data were extracted automatically from electronic medical records.

### Outcomes

The primary endpoint was all-cause mortality within 90 days of the treatment initiation in the propensity score matched population. Secondary endpoints comprised 30-day all-cause mortality, emergence of carbapenem resistance, development of acute respiratory distress syndrome (ARDS) within 7 days, initiation of extracorporeal membrane oxygenation (ECMO) within 7 days, and ICU and hospital length of stay following the index date.

ARDS was classified according to a modified Berlin definition as a PaO_2_/FiO_2_ ratio ≤300 mmHg within 7 days of treatment start, acknowledging that radiographic confirmation and exclusion of cardiogenic edema were unavailable [15].

Baseline pathogens were identified from microbiological cultures of blood, urine, and respiratory tract specimens, including bronchoalveolar lavage fluid, endotracheal aspirates, and sputum, which were obtained as part of routine ICU diagnostics. The category “unknown infection source” reflected patients with microbiological sampling but without pathogen detection. This designation therefore represents culture-negative infection rather than missing data and reflects the clinical information that was available to treating physicians. Pathogens were classified as carbapenem-resistant if the antibiogram indicated resistance to any of the following: meropenem, ertapenem, imipenem, meropenem/vaborbactam, or imipenem/relebactam. Antimicrobial susceptibility testing (AST) was performed according to routine diagnostic procedures, following the guidelines of the European Committee on Antimicrobial Susceptibility Testing (EUCAST). Emergence of resistance was defined as the detection of an MDR pathogen in any culture obtained 5–14 days after treatment initiation. Multidrug resistance (MDR) was defined according to European Centre for Disease Prevention and Control (ECDC) criteria [16]. During the follow-up period, we evaluated the potential detection bias related to unequal sampling frequency for resistance emergence by assessing the number of follow-up culture samples obtained. Fungal and viral pathogens were excluded.

We included the occurrence of acute kidney injury within 30 days as exploratory safety endpoint. Acute kidney injury was defined and staged according to the KDIGO (Kidney Disease: Improving Global Outcomes) creatinine-based criteria. Baseline serum creatinine was defined as the lowest value within ±48 h of the index date. AKI Stage I was defined as an increase in serum creatinine ≥0.3 mg/dL within 48 h or 1.5–1.9 times within 7 days; Stage II as 2.0–2.9 times baseline; and Stage III as ≥3.0 times baseline or an absolute creatinine ≥4.0 mg/dL.

### Data sources and variables

Clinical information was obtained from the hospital’s centralized electronic medical records and included patient demographics (age and sex), comorbidities, Sequential Organ Failure Assessment (SOFA) score, type of ICU at initial admission (medical or surgical), and ICU interventions such as mechanical ventilation, vasopressor therapy, corticosteroid use, antibiotic administration, and ECMO use. All time-dependent variables were referenced to the index date (initiation of meropenem treatment). Antimicrobial therapy prior to the index date, as well as key clinical parameters, such as body temperature and PaO_2_/FiO_2_ ratio measured immediately before or at the time of treatment initiation, were considered baseline variables.

Vital status at 30 and 90 days after treatment initiation was obtained from our institution’s electronic medical records, which capture all in-hospital deaths as well as deaths recorded in other hospitals within the national network. Out-of-hospital deaths after discharge (e.g., those occurring at home or abroad) were not systematically captured.

### Propensity score matching

Because the number of patients receiving high-dose meropenem substantially exceeded those receiving standard-dose meropenem, we performed propensity score matching at a 2:1 ratio using the *MatchIt* (version 4.4.0) package in R. We estimated propensity scores with a logistic regression model that included covariates based on their known or possible associations with treatment allocation and clinical outcomes. These covariates included age, sex, admission year, estimated glomerular filtration rate category (eGFR, according to the MDRD equation [17], calculated using creatinine values from routine labs that were closest available before initiation of meropenem therapy), carbapenem resistance at baseline, use of extracorporeal membrane oxygenation (ECMO) at the time of the index date, sequential organ failure assessment (SOFA) score category, treatment with antibiotics prior to the index date, source of infection, and comorbidities (including respiratory diseases, peripheral artery diseases, and chronic kidney disease). To balance baseline characteristics between treatment groups, nearest-neighbor matching was performed in a 2:1 ratio without replacement, using the logit of the propensity score as the metric for determining matches. No influential data points were detected on visual inspection (Supplementary Fig. S1). The equivalence of covariates between groups was assessed by calculating standardized mean differences, with results displayed in love plots (Supplementary Fig. S2); differences below 0.1 were deemed indicative of sufficient balance. To check for problematic multicollinearity among covariates (Supplementary Fig. S3), variance inflation factors (VIFs) were computed. All VIFs were below 5, suggesting no substantial issues with collinearity (Supplementary Fig. S1).

### Statistical analysis and sample size considerations

Baseline characteristics were summarized using means with standard deviations, medians with interquartile ranges, or frequencies with percentages, depending on the data type. The primary analysis utilized the propensity score matched dataset. For binary outcomes, weighted logistic regression models were employed, incorporating the same covariates as in the propensity score model. Additional covariate adjustments were applied post-matching to address any remaining imbalances. Absolute risk differences with 95% confidence intervals (CIs) were calculated using marginal effects models adjusted for covariates. Continuous outcomes were evaluated with linear regression models, applying the same covariate adjustments. Unadjusted models, using only the treatment indicator as a predictor, were also fitted on the complete unmatched dataset for comparison. Subgroup analyses for the primary outcome explored differences across categories such as sex (female vs. male), age (<65 years vs. ≥65 years), timing of treatment initiation (within 2 days of ICU admission vs. later), ICU type (medical vs. surgical), use of other antimicrobial agents at baseline, catecholamine requirement at treatment initiation, renal function (eGFR ≥50 vs. <50 mL/min/1.73 m^2^), carbapenem susceptibility (intermediate or resistant per EUCAST criteria), and time period (before 2020 [pre-COVID-19], 2020–2022 [COVID-19 era before Omicron], and after 2022 [Omicron era]). All statistical analyses and visualizations were conducted using R (version 4.1.2, 2021, Vienna, Austria) and RStudio (RStudio Team, 2020).

## Results

### Study population

We screened 33 578 adult critically ill patients with an ICU length of stay of at least 3 days for eligibility (Supplementary Figure S4). After excluding ineligible patients, the overall cohort comprised 4210 patients, of which 3638 received high-dose meropenem and 572 received standard-dose meropenem therapy. After 2:1 propensity score matching, the matched cohort comprised a total of 1716 patients, with 1144 patients in the high-dose and 572 patients in the standard-dose group.

Table 1 provides an overview of the study population before and after propensity score matching, which resulted in good covariate balance (Table 1 and Supplementary Fig. S2). The median age was 62 (IQR, 49–72) years in the matched cohort, and 778 (45.3%) patients were female. Meropenem was initiated after a median of 4 (IQR, 1–9) days in the high-dose and standard-dose group after ICU admission. Infection source and treatment duration were well balanced between both groups. Supplementary Table S1 summarizes the antimicrobial drug classes that were administered within 48 h prior to initiation of meropenem therapy. Supplementary Table S2 provides an overview of pathogens identified at baseline. As shown in Supplementary Table S3, the sampling frequency during the follow-up period was similar between the high-dose and standard-dose groups.

**Table 1:** Baseline patient characteristics before and after matching.

	Overall cohort				Matched cohort			
	Overall	High-Dose	Standard-dose	p	Overall	High-Dose	Standard-dose	p
n	4210	3638	572		1716	1144	572	
Age, years (median [IQR])	61 [49, 70]	61 [50, 70]	62 [49, 72]	0.419	62 [49, 72]	61 [49, 71]	62 [49, 72]	0.845
Female sex (%)	1495 (35.5)	1231 (33.8)	264 (46.2)	<0.001	778 (45.3)	514 (44.9)	264 (46.2)	0.668
Infection source (%)				<0.001				0.91
Blood	593 (14.1)	526 (14.5)	67 (11.7)		195 (11.4)	128 (11.2)	67 (11.7)	
Lung	1591 (37.8)	1397 (38.4)	194 (33.9)		579 (33.7)	385 (33.7)	194 (33.9)	
Urine	199 (4.7)	156 (4.3)	43 (7.5)		121 (7.1)	78 (6.8)	43 (7.5)	
Unknown	1827 (43.4)	1559 (42.9)	268 (46.9)		821 (47.8)	553 (48.3)	268 (46.9)	
Total daily meropenem dose (median [IQR])	6.16 [4.85, 6.40]	6.21 [5.74, 6.46]	3.28 [2.62, 4.22]	<0.001	5.82 [3.72, 6.30]	6.21 [5.82, 6.43]	3.28 [2.62, 4.22]	<0.001
Total duration of meropenem treatment, days (median [IQR])	7.46 [4.41, 11.67]	7.42 [4.33, 11.67]	7.50 [4.62, 11.51]	0.894	7.50 [4.33, 11.78]	7.56 [4.31, 12.00]	7.50 [4.62, 11.51]	0.966
Carbapenem-resistance at baseline (%)	460 (10.9)	387 (10.6)	73 (12.8)	0.149	220 (12.8)	147 (12.8)	73 (12.8)	1
Days from ICU admission to index date (median [IQR])	5.00 [1.00, 9.00]	5.00 [1.00, 9.00]	4.00 [1.00, 10.00]	0.25	4.00 [1.00, 9.00]	5.00 [1.00, 9.00]	4.00 [1.00, 10.00]	0.726
Geographical origin (%)				0.046				0.751
Africa	19 (0.5)	17 (0.5)	2 (0.3)		8 (0.5)	6 (0.5)	2 (0.3)	
Europe	3529 (83.8)	3025 (83.2)	504 (88.1)		1499 (87.4)	995 (87.0)	504 (88.1)	
Middle East or Asia	69 (1.6)	60 (1.6)	9 (1.6)		24 (1.4)	15 (1.3)	9 (1.6)	
North or South America	15 (0.4)	14 (0.4)	1 (0.2)		7 (0.4)	6 (0.5)	1 (0.2)	
Unknown	578 (13.7)	522 (14.3)	56 (9.8)		178 (10.4)	122 (10.7)	56 (9.8)	
Time period of admission (%)				<0.001				0.081
2014/15	674 (16.0)	527 (14.5)	147 (25.7)		384 (22.4)	237 (20.7)	147 (25.7)	
2016/17	793 (18.8)	674 (18.5)	119 (20.8)		422 (24.6)	303 (26.5)	119 (20.8)	
2018/19	539 (12.8)	467 (12.8)	72 (12.6)		220 (12.8)	148 (12.9)	72 (12.6)	
2020/21	1066 (25.3)	946 (26.0)	120 (21.0)		356 (20.7)	236 (20.6)	120 (21.0)	
2022/23	1032 (24.5)	927 (25.5)	105 (18.4)		303 (17.7)	198 (17.3)	105 (18.4)	
2024	106 (2.5)	97 (2.7)	9 (1.6)		31 (1.8)	22 (1.9)	9 (1.6)	
ICU type				0.001				0.384
Surgical	2234 (53.1)	1968 (54.1)	266 (46.5)		825 (48.1)	559 (48.9)	266 (46.5)	
Medical	1976 (46.9)	1670 (45.9)	306 (53.5)		891 (51.9)	585 (51.1)	306 (53.5)	
Comorbidities								
Endocrine, nutritional, and metabolic diseases (%)	692 (16.4)	568 (15.6)	124 (21.7)	<0.001	338 (19.7)	214 (18.7)	124 (21.7)	0.163
Diabetes (%)	569 (13.5)	485 (13.3)	84 (14.7)	0.415	253 (14.7)	169 (14.8)	84 (14.7)	1
Chronic kidney disease (%)	440 (10.5)	355 (9.8)	85 (14.9)	<0.001	249 (14.5)	164 (14.3)	85 (14.9)	0.827
Diseases of the digestive system (%)	805 (19.1)	689 (18.9)	116 (20.3)	0.483	345 (20.1)	229 (20.0)	116 (20.3)	0.949
Hypertensive disease (%)	3367 (80.0)	2914 (80.1)	453 (79.2)	0.656	1358 (79.1)	905 (79.1)	453 (79.2)	1
Ischemic heart disease (%)	1559 (37.0)	1348 (37.1)	211 (36.9)	0.976	617 (36.0)	406 (35.5)	211 (36.9)	0.606
Cerebrovascular disease (%)	259 (6.2)	217 (6.0)	42 (7.3)	0.237	120 (7.0)	78 (6.8)	42 (7.3)	0.763
Arterial disease (%)	349 (8.3)	293 (8.1)	56 (9.8)	0.187	171 (10.0)	115 (10.1)	56 (9.8)	0.932
Neoplasms (%)	555 (13.2)	481 (13.2)	74 (12.9)	0.904	231 (13.5)	157 (13.7)	74 (12.9)	0.708
Diseases of the respiratory system (%)	1038 (24.7)	867 (23.8)	171 (29.9)	0.002	511 (29.8)	340 (29.7)	171 (29.9)	0.985
Extracorporeal membrane oxygenation (ECMO) at index date (%)	289 (6.9)	265 (7.3)	24 (4.2)	0.009	72 (4.2)	48 (4.2)	24 (4.2)	1
Sequential Organ Failure Assessment (SOFA) score (median [IQR])	8.00 [5.00, 11.00]	8.00 [5.00, 11.00]	8.00 [5.00, 11.00]	0.81	8.00 [5.00, 11.00]	8.00 [5.00, 12.00]	8.00 [5.00, 11.00]	0.848
PaO2/FiO2 (%)				0.07				0.29
100 mmHg or less	787 (18.7)	688 (18.9)	99 (17.3)		314 (18.3)	215 (18.8)	99 (17.3)	
100 to 199 mmHg	1238 (29.4)	1072 (29.5)	166 (29.0)		533 (31.1)	367 (32.1)	166 (29.0)	
200 to 299 mmHg	508 (12.1)	424 (11.7)	84 (14.7)		227 (13.2)	143 (12.5)	84 (14.7)	
300 mmHg or more	169 (4.0)	138 (3.8)	31 (5.4)		76 (4.4)	45 (3.9)	31 (5.4)	
Not available	1508 (35.8)	1316 (36.2)	192 (33.6)		566 (33.0)	374 (32.7)	192 (33.6)	
Fever (%)				<0.001				0.251
37.5 °C or less	734 (17.4)	599 (16.5)	135 (23.6)		376 (21.9)	241 (21.1)	135 (23.6)	
37.6 to 37.9 °C	475 (11.3)	400 (11.0)	75 (13.1)		231 (13.5)	156 (13.6)	75 (13.1)	
38.0 to 39.9 °C	893 (21.2)	759 (20.9)	134 (23.4)		430 (25.1)	296 (25.9)	134 (23.4)	
40.0 °C or more	49 (1.2)	44 (1.2)	5 (0.9)		28 (1.6)	23 (2.0)	5 (0.9)	
Not available	2059 (48.9)	1836 (50.5)	223 (39.0)		651 (37.9)	428 (37.4)	223 (39.0)	
Vasopressor at time of index date (%)	2826 (67.1)	2455 (67.5)	371 (64.9)	0.233	1126 (65.6)	755 (66.0)	371 (64.9)	0.679
Inotrope at time of index date (%)	199 (4.7)	172 (4.7)	27 (4.7)	1	68 (4.0)	41 (3.6)	27 (4.7)	0.314
Antimicrobial treatment at time of index date (%)	2851 (67.7)	2492 (68.5)	359 (62.8)	0.007	1078 (62.8)	719 (62.8)	359 (62.8)	1
Corticosteroid treatment at time of index date (%)	979 (23.3)	825 (22.7)	154 (26.9)	0.029	400 (23.3)	246 (21.5)	154 (26.9)	0.015
Immunosuppression at time of index date (%)	395 (9.4)	339 (9.3)	56 (9.8)	0.777	178 (10.4)	122 (10.7)	56 (9.8)	0.634
Leukocytes at time of index date, 10^9^/L (mean (SD))	13.78 (9.69)	13.76 (9.81)	13.89 (8.86)	0.765	13.69 (8.55)	13.59 (8.40)	13.89 (8.86)	0.495
Procalcitonin at index culture, ng/mL (mean (SD))	7.64 (18.79)	7.84 (18.98)	6.02 (17.12)	0.156	6.10 (16.16)	6.13 (15.76)	6.02 (17.12)	0.928
eGFR, mL/min/1.73m^2^ (median (IQR))	69.37 [36.69, 102.77]	69.44 [34.92, 103.45]	69.20 [50.50, 97.65]	0.015	69.57 [45.19, 98.76]	70.20 [43.51, 98.99]	69.20 [50.50, 97.65]	0.342
eGFR category, mL/min/1.73m^2^ (%)				<0.001				0.993
Less than 15	222 (5.3)	217 (6.0)	5 (0.9)		16 (0.9)	11 (1.0)	5 (0.9)	
15 to 29	560 (13.3)	515 (14.2)	45 (7.9)		131 (7.6)	86 (7.5)	45 (7.9)	
30 to 59	1039 (24.7)	865 (23.8)	174 (30.4)		526 (30.7)	352 (30.8)	174 (30.4)	
60 to 89	810 (19.2)	653 (17.9)	157 (27.4)		479 (27.9)	322 (28.1)	157 (27.4)	
90 or higher	1579 (37.5)	1388 (38.2)	191 (33.4)		564 (32.9)	373 (32.6)	191 (33.4)	
Direct ICU admission (%)	2048 (48.6)	1797 (49.4)	251 (43.9)	0.016	784 (45.7)	533 (46.6)	251 (43.9)	0.312
Index date within 2 days from hospital admission (%)	832 (19.8)	713 (19.6)	119 (20.8)	0.538	353 (20.6)	234 (20.5)	119 (20.8)	0.916

Abbreviations: CRP, C-reactive protein; ECMO, extracorporeal membrane oxygenation; eGFR, estimated glomerular filtration rate; FiO₂, fraction of inspired oxygen; ICU, intensive care unit; PaO₂, partial pressure of arterial oxygen; SOFA, Sequential Organ Failure Assessment.

### Primary endpoint

**Fig. 1:**
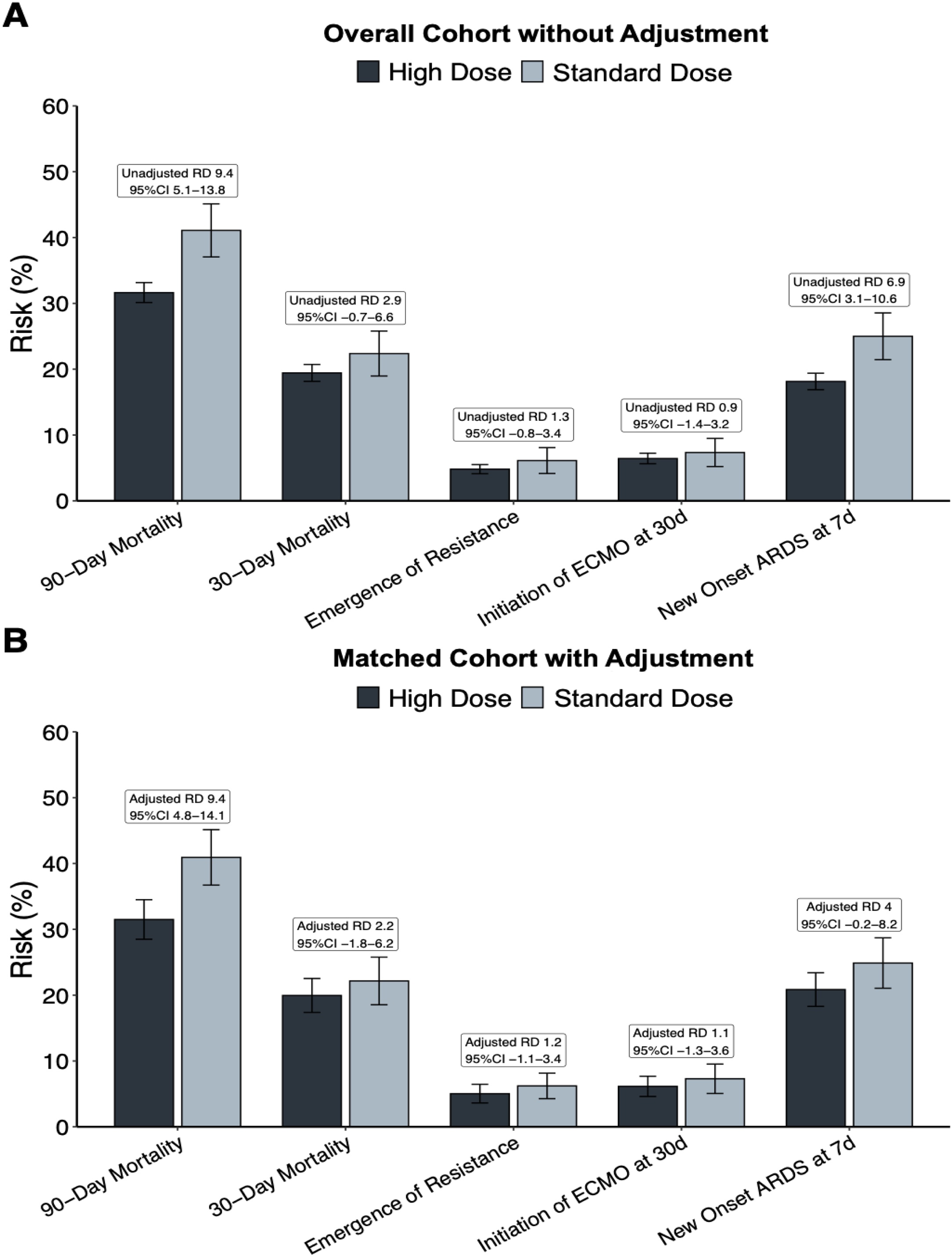
Unadjusted primary and secondary outcomes in the overall cohort (A) and adjusted outcomes in the propensity score-matched cohort (B). Abbreviations: ARDS, acute respiratory distress syndrome; ECMO, extracorporeal membrane oxygenation; RD, risk differences

**Fig. 2:**
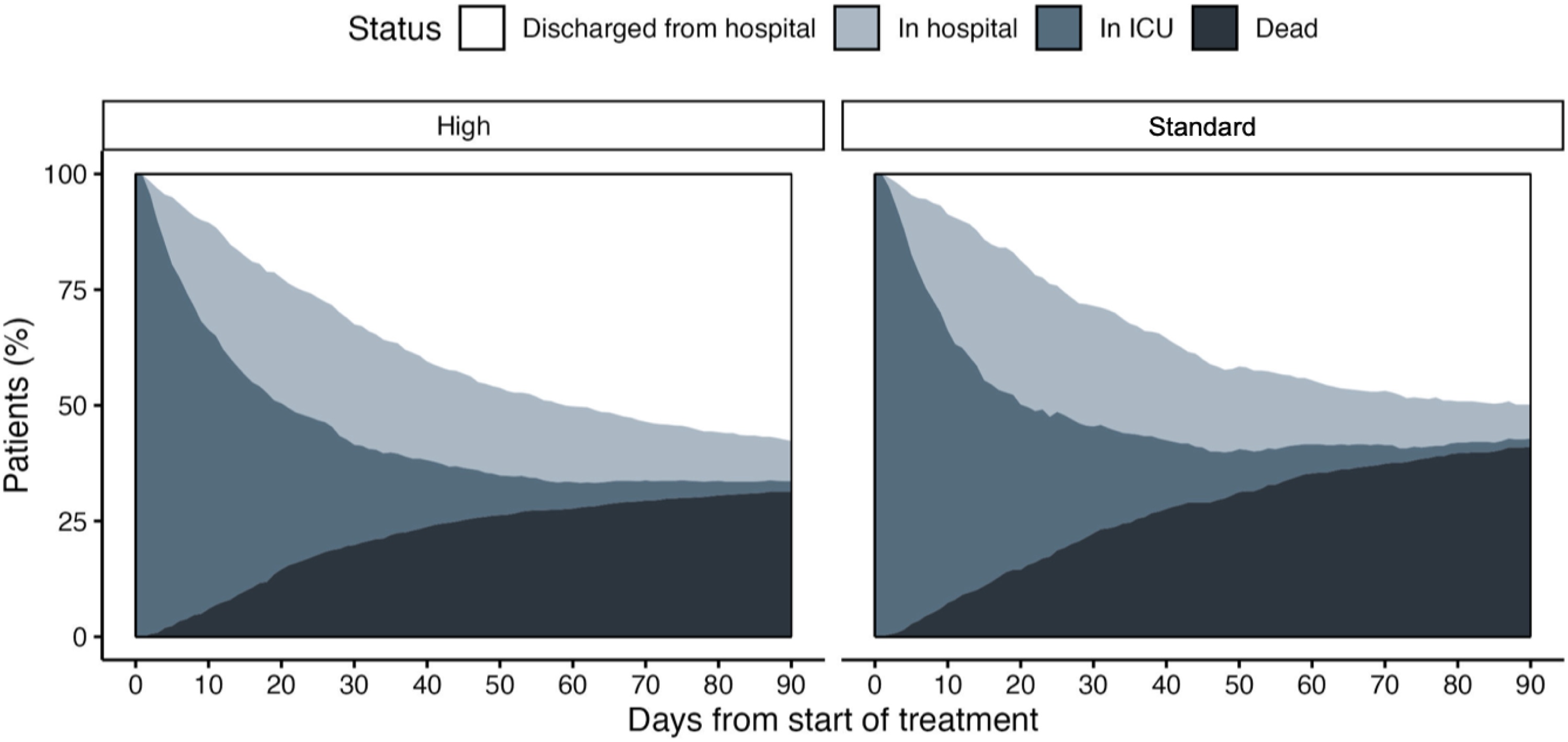
Daily patient disposition in the propensity score-matched cohort, comparing the high-dose and standard-dose meropenem groups.

In the propensity score-matched cohort, the 90-day all-cause mortality rate was significantly higher in the standard-dose meropenem group compared to the high-dose meropenem group (Table 2, Fig. 1, Fig. 2). Mortality occurred in 359 of 1144 patients (adjusted risk, 31.5%; 95% CI, 28.5–34.5) in the high-dose group and in 235 of 572 patients (adjusted risk, 40.9%; 95% CI, 36.7–45.1) in the standard-dose group, resulting in an adjusted risk difference (RD) of 9.4% (95% CI, 4.8–14.1; p < 0.001). Fig. 2 illustrates the daily patient disposition in the matched cohort.

### Secondary and safety endpoints

No statistically significant differences were observed in secondary outcomes between the high-dose and standard-dose meropenem groups (Table 2). The 30-day all-cause mortality occurred in 227 of 1144 patients (adjusted risk, 20.0%; 95% CI, 17.4–22.5) in the high-dose group and in 128 of 572 patients (adjusted risk, 22.2%; 95% CI, 18.5–25.8) in the standard-dose group, with an adjusted risk difference (RD) of 2.2% (95% CI, −1.8–6.2; p = 0.277). Resistance emergence was observed in 58 of 1144 patients (adjusted risk, 5.0%; 95% CI, 3.6–6.4) in the high-dose group and in 35 of 572 patients (adjusted risk, 6.2%; 95% CI, 4.3–8.1) in the standard-dose group, resulting in an adjusted RD of 1.2% (95% CI, −1.1–3.4). ECMO initiation at 30 days was recorded in 70 of 1144 patients (adjusted risk, 6.1%; 95% CI, 4.6–7.7) in the high-dose group and in 42 of 572 patients (adjusted risk, 7.3%; 95% CI, 5.1–9.5) in the standard-dose group, with an adjusted RD of 1.2% (95% CI, −1.3–3.6). New-onset ARDS at 7 days occurred in 238 of 1144 patients (adjusted risk, 20.8%; 95% CI, 18.3–23.4) in the high-dose group and in 143 of 572 patients (adjusted risk, 24.9%; 95% CI, 21.0–28.7) in the standard-dose group, with an adjusted RD of 4.0% (95% CI, −0.2–8.2).

**Table 2:** Primary and secondary outcomes in the overall and matched cohort.

All-cause death at 90 days								
	Overall cohort without adjustment				Matched cohort with adjustment			
	N (3638 vs 572)	Unadjusted Risk (95% CI)	Unadjusted RD (95% CI)	P	N (1144 vs 572)	Adjusted risk (95% CI)	Adjusted RD (95% CI)	P
High-dose	1151	31.6 (30.1–33.1)			359	31.5 (28.5–34.5)		
Standard-dose	235	41.1 (37.1–45.1)	9.4 (5.1–13.8)	<0.001	235	40.9 (36.7–45.1)	9.4 (4.8–14.1)	<0.001
All-cause death at 30 days								
High-dose	707	19.4 (18.1–20.7)			227	20 (17.4–22.5)		
Standard-dose	128	22.4 (19–25.8)	2.9 (−0.7–6.6)	0.114	128	22.2 (18.5–25.8)	2.2 (−1.8–6.2)	0.277
Emergence of resistance								
High-dose	175	4.8 (4.1–5.5)			58	5 (3.6–6.4)		
Standard-dose	35	6.1 (4.2–8.1)	1.3 (−0.8–3.4)	0.218	35	6.2 (4.3–8.1)	1.2 (−1.1–3.4)	0.302
Initiation of ECMO at 30 days								
High-dose	234	6.4 (5.6–7.2)			70	6.1 (4.6–7.7)		
Standard-dose	42	7.3 (5.2–9.5)	0.9 (−1.4–3.2)	0.433	42	7.3 (5.1–9.5)	1.2 (−1.3–3.6)	0.357
New onset of ARDS at 7 days								
High-dose	660	18.1 (16.9–19.4)			238	20.8 (18.3–23.4)		
Standard-dose	143	25 (21.5–28.5)	6.9 (3.1–10.6)	0	143	24.9 (21–28.7)	4 (−0.2–8.2)	0.059
Length of ICU stay after treatment start								
		Mean (95% CI)	Unadjusted Difference (95% CI)			Adjusted Mean (95% CI)	Adjusted Difference (95% CI)	
High-dose		21.7 (20.9–22.4)				21.5 (20–23)		
Standard-dose		21.4 (19.5–23.3)	−0.3 (−2.3–1.8)	0.797		21.4 (19.5–23.4)	−0.1 (−2.3–2.1)	0.946
Length of hospital stay after treatment start								
High-dose		43.5 (41.1–45.8)				44.3 (39.5–49.1)		
Standard-dose		41.8 (35.9–47.7)	−1.7 (−8–4.7)	0.611		41.7 (35.3–48.1)	−2.6 (−9.8–4.6)	0.48

The adjusted mean ICU stay was 21.5 days (95% CI, 20.0–23.0) in the high-dose group and 21.4 days (95% CI, 19.5–23.4) in the standard-dose group, with an adjusted difference of −0.1 days (95% CI, −2.3–2.1;). The adjusted mean hospital stay was 44.3 days (95% CI, 39.5–49.1) in the high-dose group and 41.7 days (95% CI, 35.3–48.1) in the standard-dose group, with an adjusted difference of −2.6 days (95% CI, −9.8–4.6).

The high-dose group showed a lower risk of any acute kidney injury (60.6%; 95% CI, 57.4–63.8) compared with the standard-dose group (68.2%; 95% CI, 64.3–72.1) (adjusted RD 7.6%; 95% CI, 3.1–12.1) (Supplementary Figure S5). This difference was primarily driven by a reduced risk of acute kidney injury stage III in the high-dose group (14.6%; 95% CI, 12.3–16.9) versus the standard-dose group (21.4%; 95% CI, 17.9–25.0) (adjusted RD 6.8%; 95% CI, 3.0–10.7).

### Subgroup analyses

**Fig. 3:**
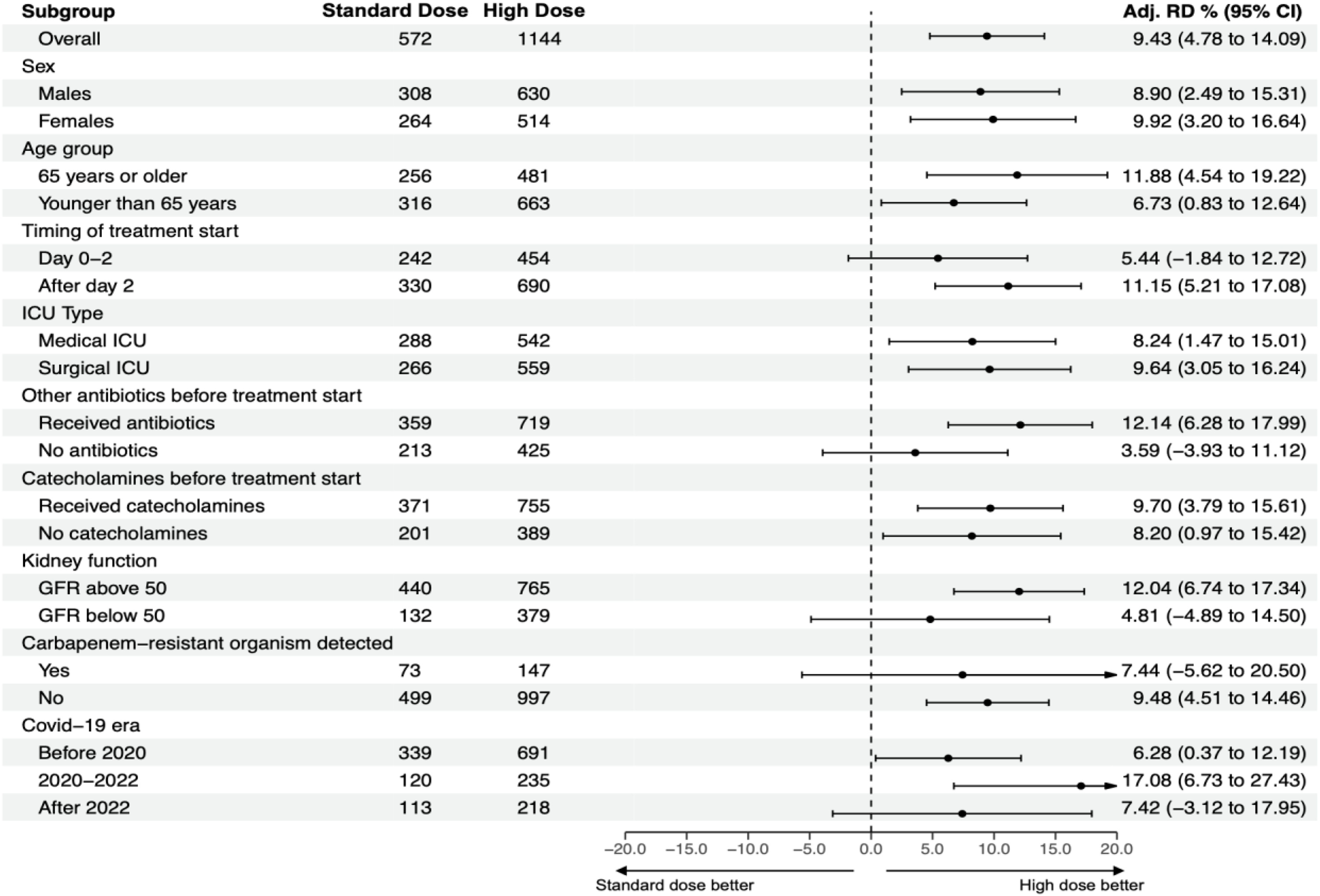
Subgroup analyses of the primary endpoint 90-day all-cause mortality. Abbreviations: GFR, glomerular filtration rate in mL/min/1.73 m^2^, ICU, intensive care unit

Subgroup analyses in the propensity score-matched cohort showed that the 90-day all-cause mortality benefit of high-dose meropenem was consistent across several subgroups (Fig. 3). Despite a consistent direction of effect, statistical significance was not reached in patients given meropenem within two days of ICU admission (adjusted RD 5.4; 95% CI, −1.8–12.7), those receiving no other antibiotics before therapy (adjusted RD 3.6; 95% CI, −3.9–11.1), individuals with an eGFR below 50 ml/min/1.73 m^2^ (adjusted RD 4.8; 95% CI, −4.9–14.5), and those with baseline carbapenem resistance (adjusted RD 7.4; 95% CI, −5.6–20.5). Supplementary Figure S6 summarizes the subgroup analyses for the secondary endpoint of 30‑day all‑cause mortality, which revealed no significant heterogeneity.

## Discussion

In this observational, propensity score matched cohort study of critically ill patients, high-dose meropenem was associated with a significant reduction in 90-day all-cause mortality compared to standard-dose regimens in the matched cohort. There were no significant differences in secondary endpoints, including 30-day all-cause mortality, initiation of ECMO, incidence of ARDS, emergence of resistance or length of hospital or ICU stay in the matched cohort. Risk of acute kidney injury was lower in the high-dose group than in the standard-dose group.

The observed mortality benefit aligns with PK/PD principles of β-lactam antibiotics. The PK/PD index that best estimates the efficacy of meropenem is the proportion of the dosing interval during which free drug concentrations exceed the minimum inhibitory concentration (*f*T_>MIC_) [18]. In critically ill patients, pathophysiological alterations, including augmented renal clearance, increased volume of distribution, and variable organ dysfunction, can reduce drug exposure, leading to suboptimal PK/PD target attainment when conventional doses are used [4]. Previous studies have shown that conventional 3 g daily dosing may result in insufficient drug exposure, thereby compromising efficacy [3,5]. Higher doses of meropenem of 6 g per day (or an equivalent dose in patients with reduced renal function) may not only be associated with a favorable PK/PD target attainment but also with improved clinical outcomes.

However, the clinical evidence to date remains limited. Lertwattanachai et al. reported that in critically ill patients with sepsis or septic shock, high-dose intermittent meropenem (6 g/day) did not improve the primary endpoint of modified SOFA score and showed similar 28-day all-cause mortality and clinical cure rates compared with standard dosing (3 g/day), despite superior PK/PD target attainment [12]. Furthermore, a small randomized trial found that high-dose versus standard-dose meropenem for ventilator-associated pneumonia caused by MDR bacteria showed no significant difference in clinical success or reduction in 28-day all-cause mortality [11]. This lack of benefit may be due the inclusion of only patients with baseline carbapenem-resistant pathogens, which aligns with our subgroup analysis showing no advantage of high-dose meropenem in these patients. In such cases, alternative therapies or combination regimens may be more appropriate than simply escalating the dose. Nevertheless, our study contributes to this evidence by suggesting that, under real-world conditions, where clinicians typically do not have access to exact MIC values or PK/PD calculations, increasing the total daily dose of meropenem to 6 g may translate into clinically meaningful mortality benefits.

In our cohort, 30-day mortality did not differ between groups, whereas high-dose therapy was associated with lower 90-day mortality. Although this association persisted across multiple sensitivity analyses and may suggest potential longer-term benefits, later outcomes remain susceptible to residual confounding and downstream care pathways, and may not exclusively reflect the direct effect of initial meropenem dosing. In our cohort, 30-day mortality showed the same direction of effect as the 90-day endpoint but did not reach statistical significance, potentially due to the lower number of early deaths and consequently reduced statistical power to detect absolute risk differences, despite a relative difference exceeding 10%. In contrast, 90-day mortality captures the cumulative impact of critical illness, complications, and late deterioration, including outcomes following prolonged organ support such as mechanical ventilation or ECMO, which can defer early mortality without altering longer-term survival. Thus, consistent numerical trends across secondary outcomes and the significant difference at 90 days suggest a trajectory that may not be fully reflected in shorter-term measures.

As an exploratory safety endpoint, we assessed acute kidney injury within 30 days using KDIGO creatinine-based criteria. AKI was frequent (>60% overall), in line with large ICU cohorts of severe infection [19]. The high-dose meropenem group showed a lower adjusted risk of any AKI (60.6% vs. 68.2%), driven primarily by fewer stage III events (14.6% vs. 21.4%). Because AKI may reflect either direct nephrotoxicity or persistent sepsis-related organ dysfunction from inadequate bacterial elimination, the observed renal safety signal with higher dosing is plausible but remains hypothesis-generating.

Our subgroup analyses provide additional insights into which patient populations may benefit the most from high-dose meropenem. For example, we observed a greater benefit in patients who initiated meropenem later in their ICU course (after day 2) rather than immediately upon admission. One possible explanation is that delayed initiation may reflect escalation after failure of other regimens, in which case higher meropenem exposure provided a therapeutic advantage against partially susceptible pathogens. Similarly, a benefit was noted in patients who had received other antibiotics prior to meropenem initiation but not in those without prior therapy, supporting the interpretation that escalation to meropenem in the context of non-response to earlier therapy may be a key scenario where higher dosing adds value. High-dose meropenem was associated with mortality reduction in patients with an eGFR >50 mL/min/1.73 m^2^ but not in those with impaired renal function. This may reflect that reduced renal clearance provides adequate drug exposure at lower doses, while higher doses could increase nephrotoxicity risk, limiting any potential benefit. Taken together, our results provide evidence that high-dose meropenem can improve survival in critically ill patients under specific conditions, particularly in those with preserved renal function and susceptible pathogens. Simultaneously, they emphasize that high-dose meropenem is not universally beneficial in certain populations according to our subgroup analyses.

We acknowledge several limitations. First, the observational design introduced the possibility of residual confounding, despite the use of propensity score matching and covariate adjustment. Furthermore, the lack of therapeutic drug monitoring TDM prevented direct measurement of meropenem exposure and PK/PD target attainment. Since therapeutic drug monitoring samples were infrequently obtained and lacked standardized timing and protocols, a reliable analysis was not feasible. Moreover, precise MICs, infection sources, and underlying pathogens were not consistently available, as is often the case even in prospective studies, making traditional PK/PD calculations unfeasible. However, this study aimed to evaluate the real-world performance of high-dose versus standard-dose meropenem in a heterogeneous ICU population, prioritizing clinical outcomes over precise PK/PD metrics. Although the 90-day all-cause mortality was significantly higher in the standard-dose group, the difference in 30-day all-cause mortality, while numerically higher in the same group, did not reach statistical significance. Notably, the 30-day all-cause mortality, emergence of resistance, and new onset of ARDS occurred less frequently in the high-dose meropenem group than in the standard-dose group, albeit without reaching statistical significance. These subtle differences may have cumulatively contributed to the significant survival signal. Finally, our study did not systematically capture out-of-hospital deaths, as vital status was derived from institutional electronic medical records and deaths recorded within the national hospital network. However, this is unlikely to bias early mortality outcomes because out-of-hospital death in critically ill patients within this period is uncommon, and any missing data would be expected to dilute, not create, mortality differences.

## Conclusions

In this propensity score matched cohort of critically ill patients, high-dose meropenem was independently associated with a statistically significant reduction in 90-day all-cause mortality. The differences in secondary endpoints of 30-day all-cause mortality, emergence of resistance, ARDS, initiation of ECMO, and length of hospital and ICU stay between high-dose and standard-dose meropenem groups were not statistically significant. High-dose therapy was associated with a lower adjusted risk of AKI, primarily driven by a reduced risk of acute kidney injury stage III.

## CRediT authorship contribution statement

FB, AJ, and MP conceived the study idea. KK performed the systematic data extraction. FB, LP, and AJ performed the statistical analysis and created figures and tables. FB and MP drafted the manuscript. All authors critically revised the manuscript and approved the final version.

## Funding

This study did not receive external funding.

## Availability of data and materials

Data will be shared upon reasonable request.

## Declaration of competing interest

None of the authors report any conflict of interests relevant to this work.
