# Supplementary material for: High-dose versus standard-dose intermittent meropenem in critically ill patients: An observational cohort study
Source: Ann Intensive Care. 2026 Jan 16;16:100022. doi: 10.1016/j.aicoj.2025.100022 (PMC12934412; doi:10.1016/j.aicoj.2025.100022)
Supplement: Supplementary file 1 [file mmc1.docx]

# SUPPLEMENTARY APPENDIX

# High-dose versus standard-dose intermittent meropenem in critically ill patients: an observational cohort study

# Supplementary Tables

**Supplementary Table 1** Prior antimicrobial use at baseline in the matched cohort

|  | **Overall** | **High** | **Standard** | **p** |
| --- | --- | --- | --- | --- |
| **n** | 1716 | 1144 | 572 |  |
| **Received any antibiotic treatment** | 1078 (62.8) | 719 (62.8) | 359 (62.8) | 1.000 |
| **Aminopenicillins** | 131 (7.6) | 98 (8.6) | 33 (5.8) | 0.05 |
| **Other Penicillins** | 8 (0.5) | 5 (0.4) | 3 (0.5) | 1.000 |
| **Extended Spectrum Penicillins** | 551 (32.1) | 360 (31.5) | 191 (33.4) | 0.454 |
| **1st Gen Cephalosporins** | 35 (2.0) | 29 (2.5) | 6 (1.0) | 0.061 |
| **2nd Gen Cephalosporins** | 36 (2.1) | 25 (2.2) | 11 (1.9) | 0.858 |
| **3rd Gen Cephalosporins** | 53 (3.1) | 43 (3.8) | 10 (1.7) | 0.034 |
| **4th Gen Cephalosporins** | 97 (5.7) | 59 (5.2) | 38 (6.6) | 0.252 |
| **5th Gen Cephalosporins** | 1 (0.1) | 1 (0.1) | 0 (0.0) | 1.000 |
| **Carbapenems** | 3 (0.2) | 2 (0.2) | 1 (0.2) | 1.000 |
| **Fluoroquinolones** | 62 (3.6) | 33 (2.9) | 29 (5.1) | 0.032 |
| **Aminoglycosides** | 87 (5.1) | 60 (5.2) | 27 (4.7) | 0.726 |
| **Macrolides** | 112 (6.5) | 76 (6.6) | 36 (6.3) | 0.863 |
| **Clindamycin** | 34 (2.0) | 20 (1.7) | 14 (2.4) | 0.426 |
| **Tetracyclines** | 21 (1.2) | 8 (0.7) | 13 (2.3) | 0.010 |
| **Sulfonamides** | 57 (3.3) | 45 (3.9) | 12 (2.1) | 0.063 |
| **Glycopeptides** | 58 (3.4) | 34 (3.0) | 24 (4.2) | 0.238 |
| **Oxazolidinones** | 88 (5.1) | 64 (5.6) | 24 (4.2) | 0.262 |
| **Rifamycins** | 1 (0.1) | 1 (0.1) | 0 (0.0) | 1.000 |
| **Nitroimidazoles** | 69 (4.0) | 43 (3.8) | 26 (4.5) | 0.515 |
| **Aztreonam** | 24 (1.4) | 14 (1.2) | 10 (1.7) | 0.513 |
| **Polymyxins** | 18 (1.0) | 11 (1.0) | 7 (1.2) | 0.802 |
| **Fosfomycin** | 50 (2.9) | 39 (3.4) | 11 (1.9) | 0.116 |
| **Others** | 38 (2.2) | 23 (2.0) | 15 (2.6) | 0.523 |

**Supplementary Table 2** Pathogen distribution in the matched cohort

|  | **Overall** | **High Dose** | **Standard Dose** | **p** |
| --- | --- | --- | --- | --- |
| **n** | 1716 | 1144 | 572 |  |
| **Staphylococcus spp.** |  |  |  |  |
| **Normal** | 560 (47.9) | 365 (47.5) | 195 (48.8) | 0.722 |
| **MDR** | 18 (1.5) | 12 (1.6) | 6 (1.5) | 1.000 |
| **Streptococcus spp.** |  |  |  |  |
| **Normal** | 40 (3.4) | 24 (3.1) | 16 (4.0) | 0.539 |
| **MDR** | 0 (0) | 0 (0) | 0 (0) | NA |
| **Enterococcus spp.** |  |  |  |  |
| **Normal** | 434 (37.1) | 282 (36.7) | 152 (38.0) | 0.702 |
| **MDR** | 17 (1.5) | 14 (1.8) | 3 (0.8) | 0.233 |
| **Serratia spp.** |  |  |  |  |
| **Normal** | 78 (6.7) | 47 (6.1) | 31 (7.8) | 0.347 |
| **MDR** | 7 (0.6) | 2 (0.3) | 5 (1.2) | 0.093 |
| **Pseudomonas spp.** |  |  |  |  |
| **Normal** | 279 (23.9) | 185 (24.1) | 94 (23.5) | 0.889 |
| **MDR** | 160 (13.7) | 105 (13.7) | 55 (13.8) | 1.000 |
| **Acinetobacter spp.** |  |  |  |  |
| **Normal** | 30 (2.6) | 21 (2.7) | 9 (2.2) | 0.765 |
| **MDR** | 19 (1.6) | 13 (1.7) | 6 (1.5) | 1.000 |
| **Klebsiella spp.** |  |  |  |  |
| **Normal** | 299 (25.6) | 204 (26.5) | 95 (23.8) | 0.336 |
| **MDR** | 58 (5.0) | 42 (5.5) | 16 (4.0) | 0.342 |
| **Escherichia coli** |  |  |  |  |
| **Normal** | 222 (19.0) | 148 (19.2) | 74 (18.5) | 0.818 |
| **MDR** | 52 (4.4) | 38 (4.9) | 14 (3.5) | 0.325 |
| **Enterobacter spp.** |  |  |  |  |
| **Normal** | 183 (15.7) | 126 (16.4) | 57 (14.2) | 0.385 |
| **MDR** | 87 (7.4) | 58 (7.5) | 29 (7.2) | 0.950 |
| **Other** | 646 (55.3) | 424 (55.1) | 222 (55.5) | 0.955 |
| **Normal** |  |  |  |  |
| **MDR** | 35 (3.0) | 24 (3.1) | 11 (2.8) | 0.863 |

**Supplementary Table 3** Frequency of late microbiological sampling in the matched cohort

|  | **Overall** | **High Dose** | **Standard Dose** | **p** |
| --- | --- | --- | --- | --- |
| **Any late Culture** | 879 (51.2) | 583 (51.0) | 296 (51.7) | 0.798 |
| **Any late Blood Culture** | 647 (37.7) | 428 (37.4) | 219 (38.3) | 0.765 |
| **Any late Urine Culture** | 284 (16.6) | 182 (15.9) | 102 (17.8) | 0.346 |
| **Any late Respiratory Culture** | 460 (26.8) | 304 (26.6) | 156 (27.3) | 0.802 |

# Supplementary Figures


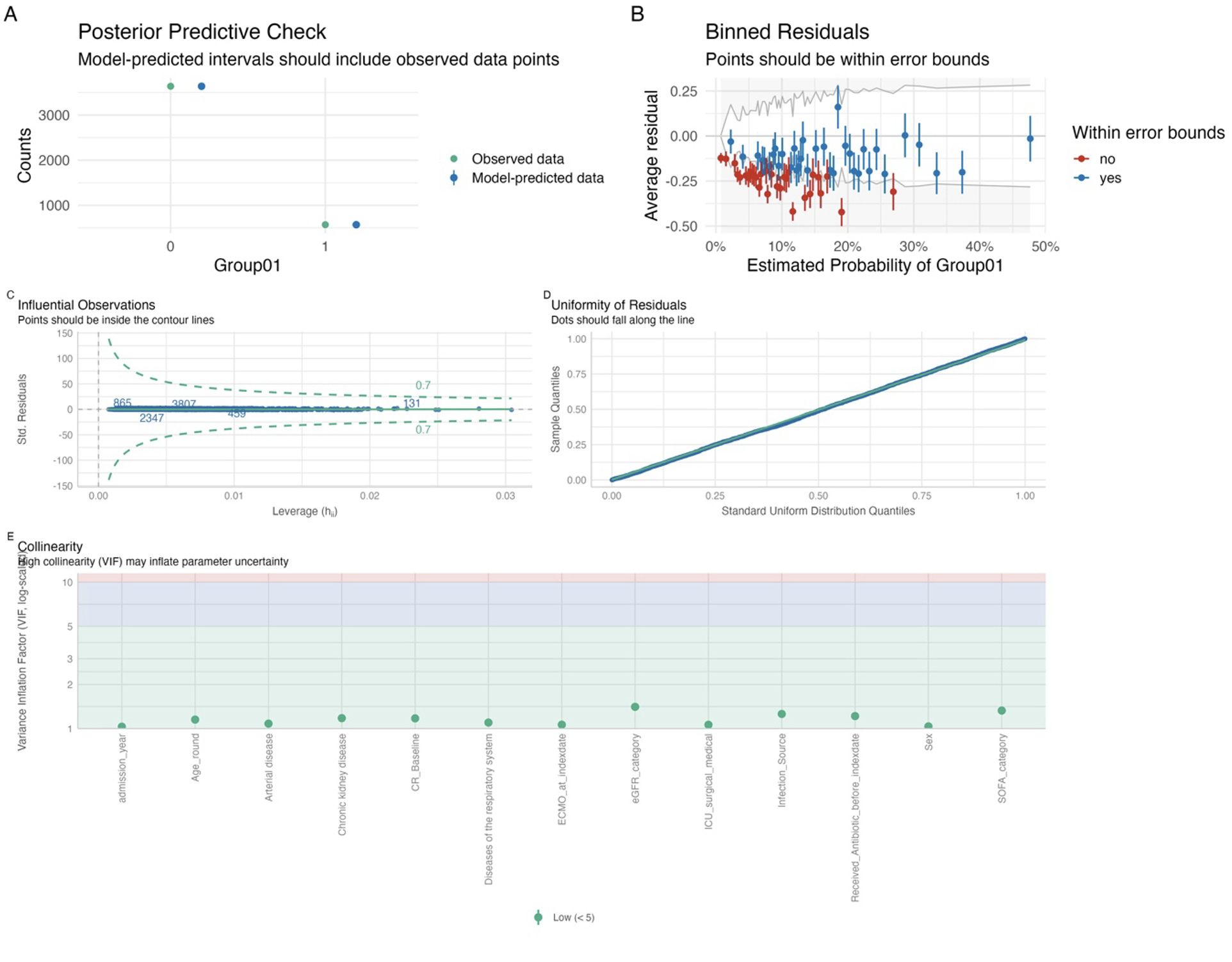


**Supplementary Figure 1** Diagnostic plots of the generalized linear model used for the overall cohort

**
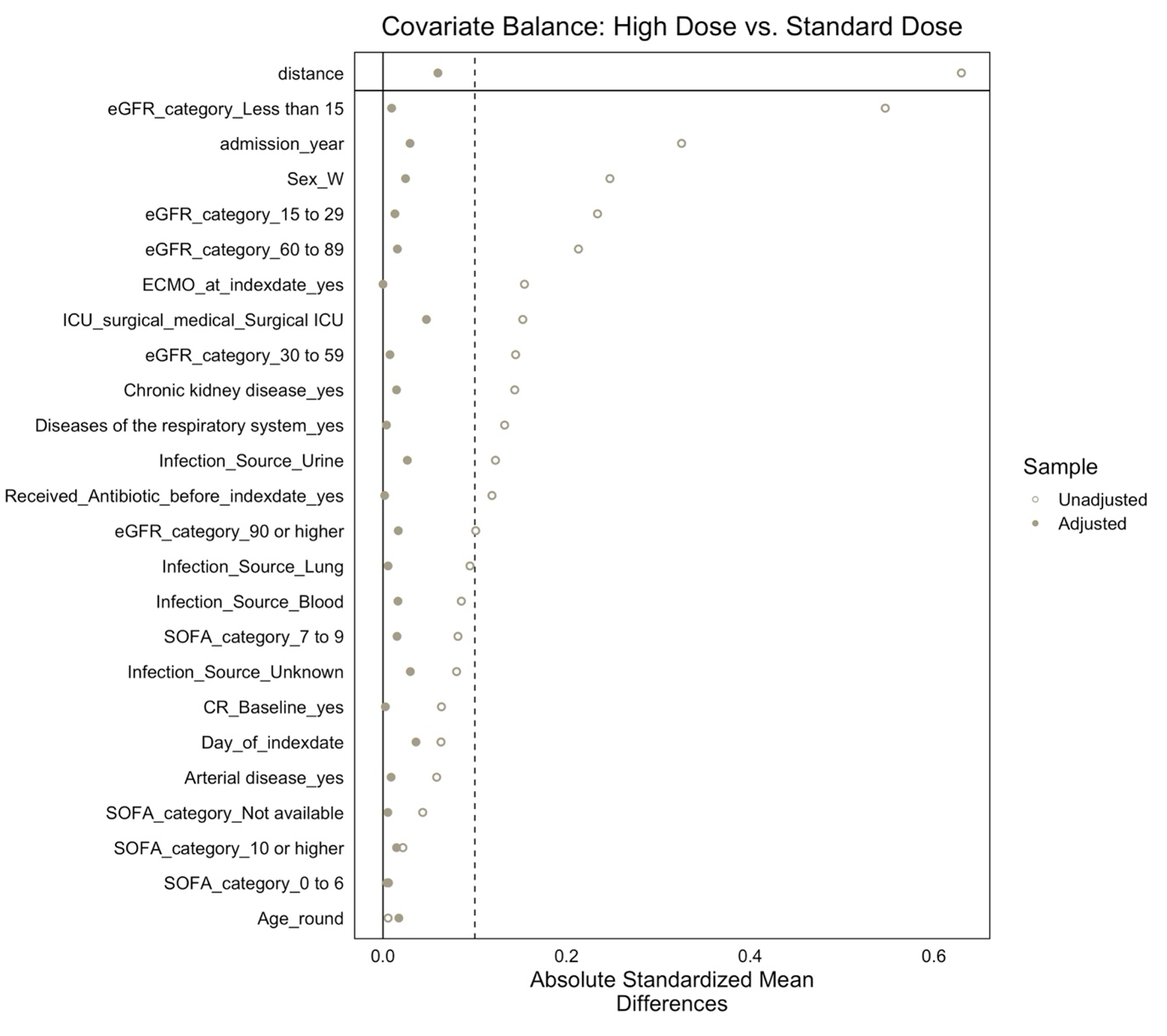
**

**Supplementary Figure 2** Assessment of covariate balance before (empty circles) and after (full circles) propensity-score matching.

The vertical dashed lines indicate the usual threshold for acceptable covariate balance, which corresponds to an absolute standardized mean difference (SMD) of 0.1.

**
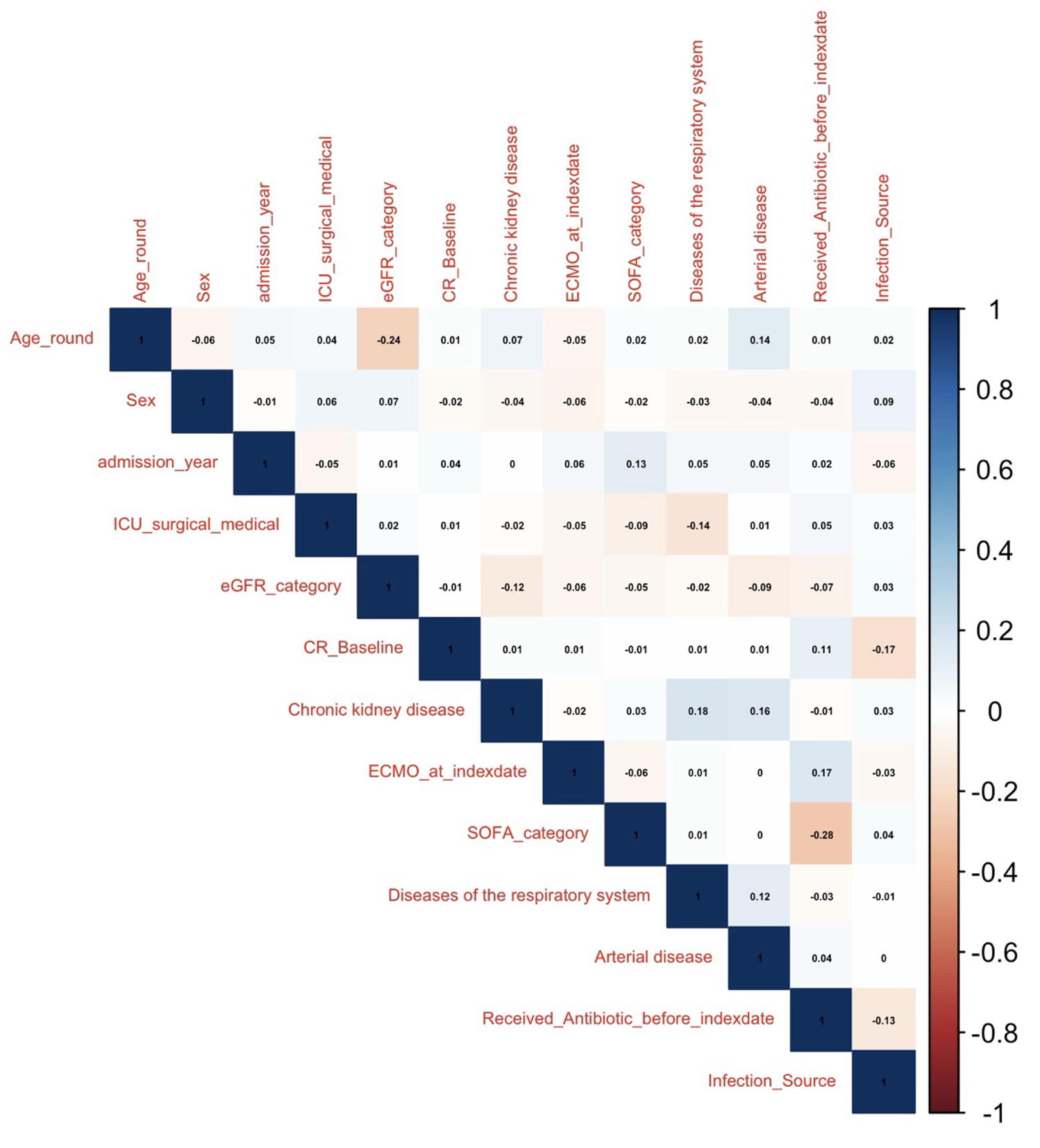
**

**Supplementary Figure 3** Multicollinearity of covariates used in the weighting and the generalized linear model for the overall cohort


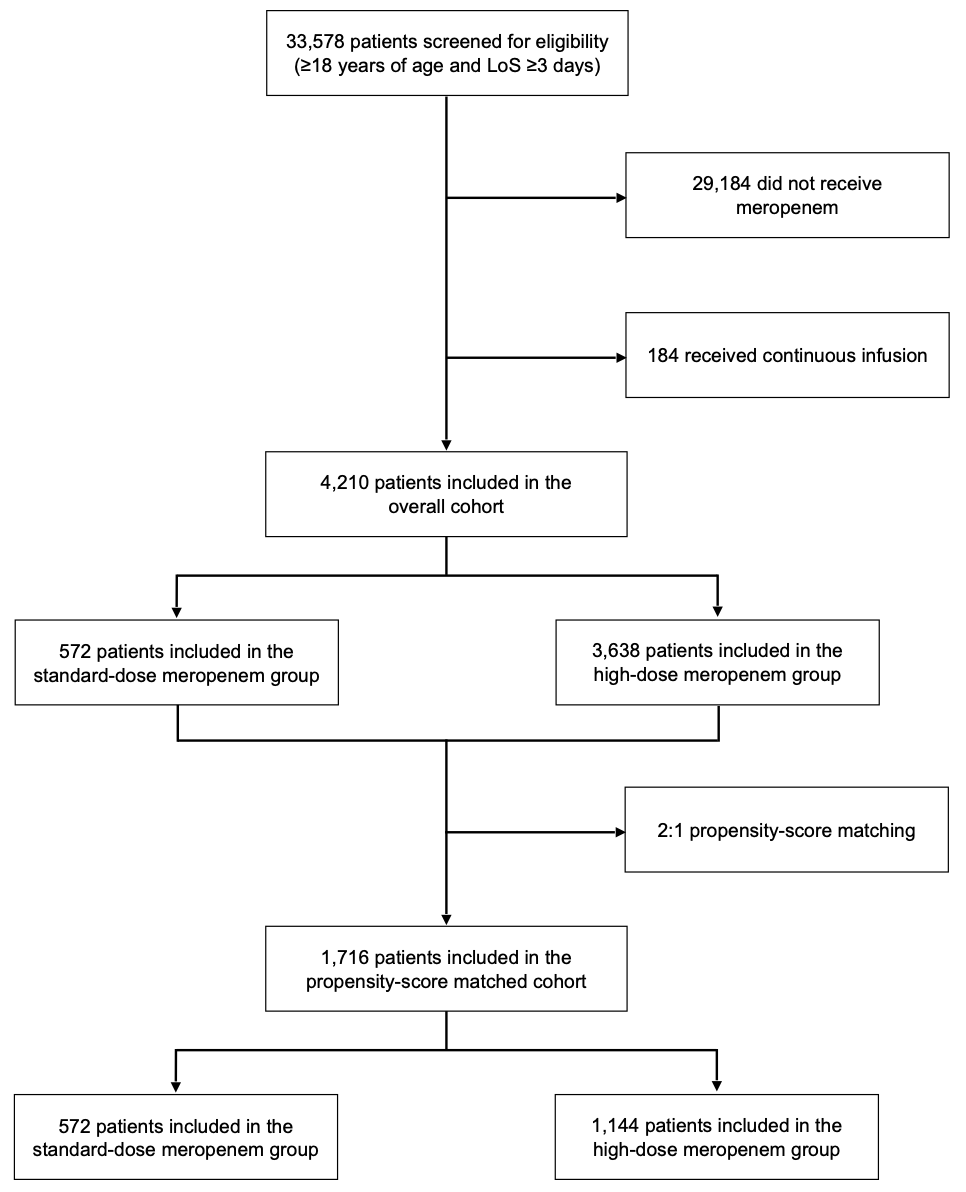


**Supplementary Figure 4** Flow chart of the study population


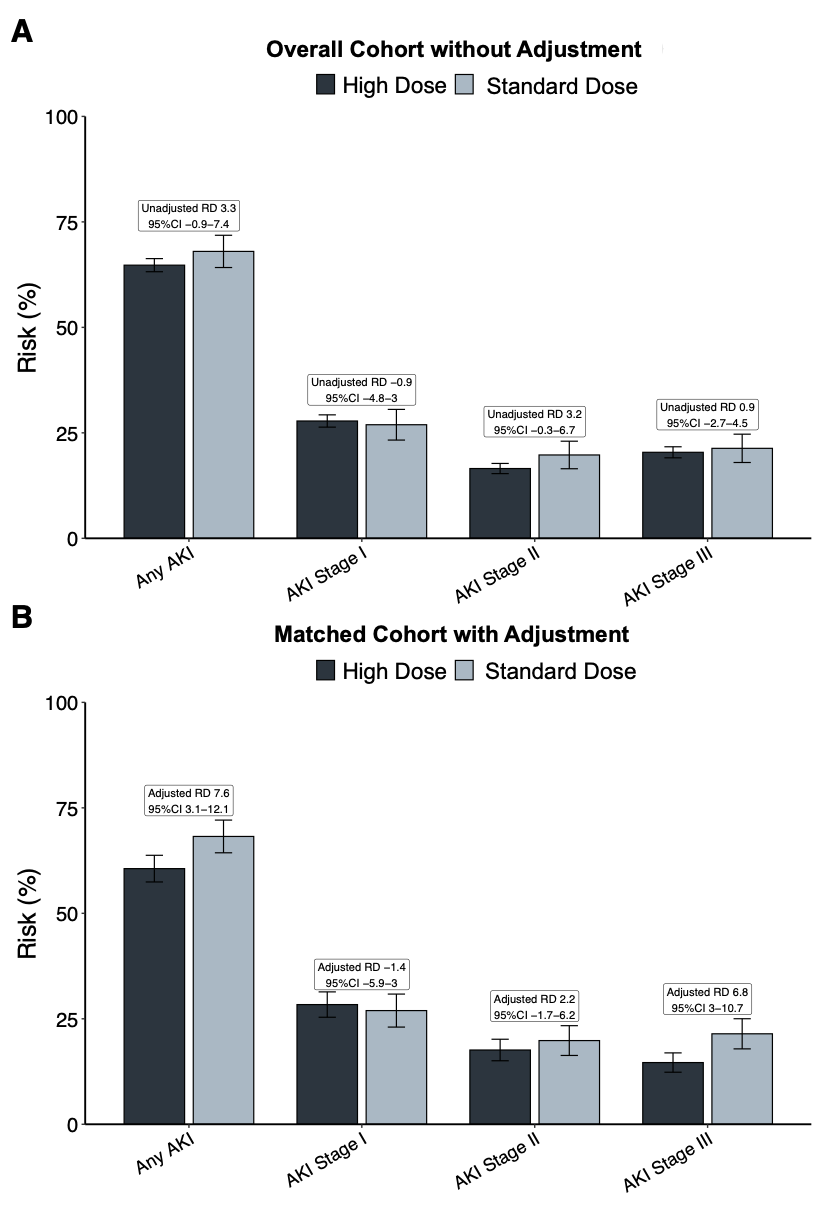


**Supplementary Figure 5** Occurrence of acute kidney injuries in the matched cohort.


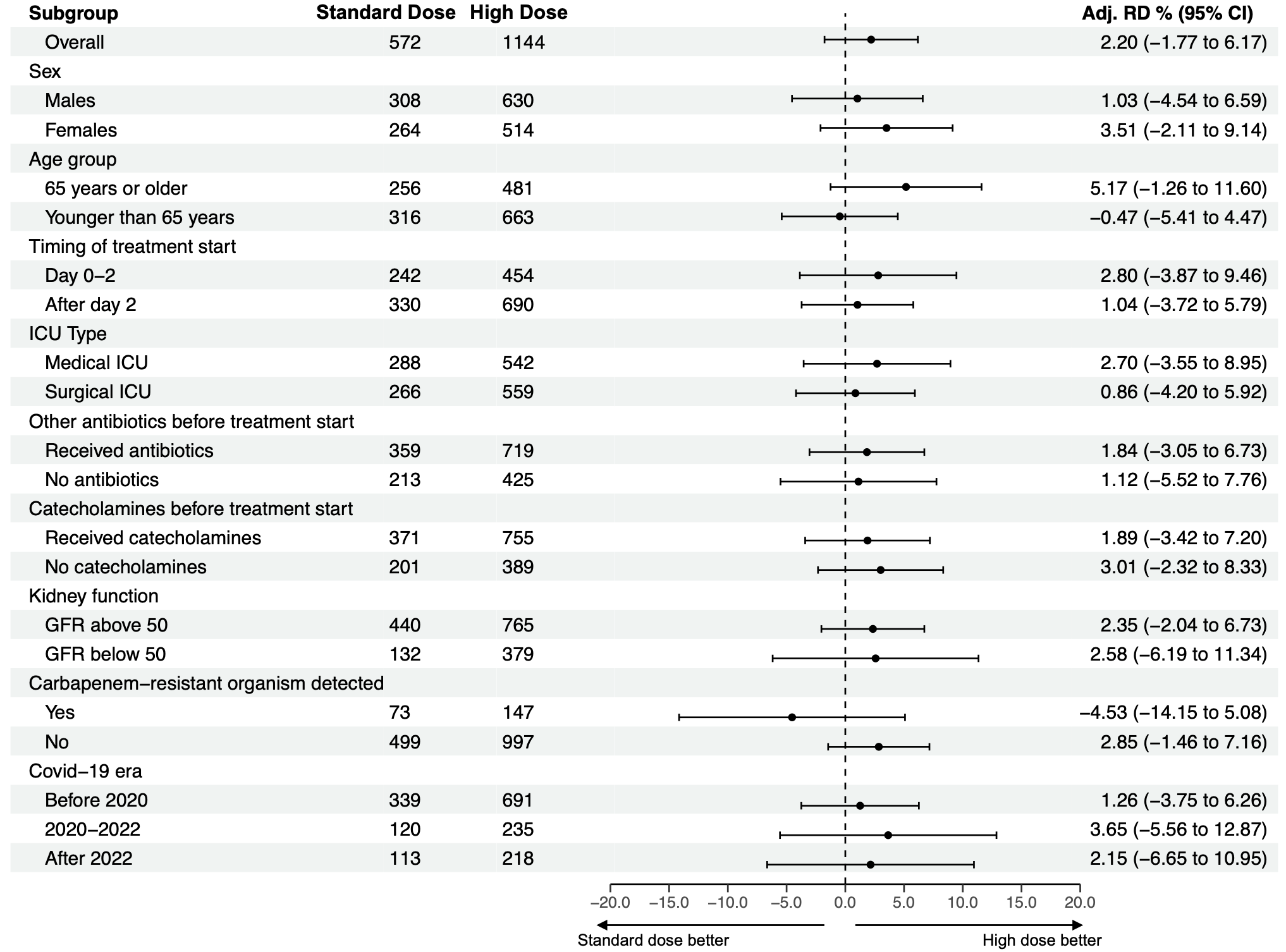


**Supplementary Figure 6** Subgroup analyses for 30-day all-cause mortality
